# The Relationship of Dietary Pattern and Genetic Risk Score with the Incidence of Dyslipidemia: 14-Year Follow-Up Cohort Study

**DOI:** 10.3390/nu12123840

**Published:** 2020-12-16

**Authors:** Seon-Joo Park, Myung-Sunny Kim, Sang-Woon Choi, Hae-Jeung Lee

**Affiliations:** 1 Department of Food and Nutrition, College of BioNano Technoloyg, Gachon University, Gyeonggi 13120, Korea; chris0825@hanmail.net; 2 Research Group of Healthcare, Korea Food Research Institute, Wanju 55365, Korea; truka@kfri.re.kr; 3 Department of Food Biotechnology, Korea University of Science and Technology, Daejeon 34113, Korea; 4 Chaum Life Center, CHA University, Seoul 06062, Korea

**Keywords:** diet, dyslipidemia, genetics, cohort study

## Abstract

This study was conducted to investigate the relationship between dietary pattern and genetic risk score (GRS) for dyslipidemia risk among Korean adults. Hypercholesterolemia and hypertriglyceridemia defined as total cholesterol ≥240 mg/dL and triglyceride ≥200 mg/dL or use dyslipidemia medication. The GRS was calculated by summing the risk alleles of the selected seven single-nucleotide polymorphisms related to dyslipidemia. Dietary patterns were identified by principal component analysis based on the frequency of 36 food groups, “whole grain and soybean products” pattern, “meat, fish and vegetables” pattern, and “bread and noodle” pattern were identified. Hazard ratios (HRs) and 95% confidence intervals (CIs) were estimated using the multivariate Cox proportional hazards regression model. High intake of a “whole grain and soybean products” pattern decreased risks of hypercholesterolemia (HR: 0.82, 95% CI: 0.72–0.93, *p* for trend = 0.0006) and hypertriglyceridemia (HR: 0.85, 95% CI: 0.75–0.97, *p* for trend = 0.0344). In the highest tertile of GRS, the “whole grain and soybean products” pattern was inversely related to hypercholesterolemia risk. Therefore, for people with genotypes that can cause hypercholesterolemia, eating whole grains and soybean products may have a meaningful response, these results could be utilized for genome-based nutrition management.

## 1. Introduction

Dyslipidemia has been closely linked to the development of coronary artery disease (CAD) and is a modifiable risk factor using dietary therapy [1]. According to a 2015 report from the Korean Society of Lipidology and Atherosclerosis, 47.8% of Korean adults over 30 years were diagnosed with dyslipidemia [2]. Several contributing factors including low physical activity, smoking, and unhealthy diet have been linked to dyslipidemia and subsequently cardiovascular diseases (CVD) worldwide [3]. Recently, dietary pattern approach is widely used in nutrition epidemiology research rather than the conventional emphasis on individual foods, nutrients or dietary components analysis [4]. Dietary patterns are thought to be associated with CVD and dyslipidemia. Vegetarian dietary patterns have beneficial effects on CVD [5], and adherence to healthy eating patterns have been associated with a lower risk of developing CVD in three large prospective cohort studies [6]. Additionally, several cross-sectional studies conducted to investigate an association between dietary patterns and dyslipidemia [7,8,9].

The lipid metabolism could be affected by specific genetic variation [10], common single-nucleotide polymorphisms (SNPs) modulate the individual response to the diet which could explain how gene-diet interactions affect lipid metabolism [11]. The effect of any single SNP on the risk of coronary heart disease (CHD) is however, too small, so the definition of genetic risk score (GRS) has arisen based on the combined impact of multiple SNPs leading to CHD [12]. The GRS is a simple and intuitive approach aggregating disease-related loci for predicting disease susceptibility [13]. It has been reported that GRS is associated with incident CVD in the Framingham Heart Study [14] and dyslipidemia among Brazilian [15]. Furthermore, the GRS modified the association between diet quality and dyslipidemia among Swedish cohort [16]. We hypothesized that the GRS of selected genetic variants would be related the risk of dyslipidemia and interact with dietary patterns. To the best of our knowledge, there is only a few studies have been performed to examine the association between dietary pattern and dyslipidemia in middle-aged Korean adults [17,18]. However, there was no finding that identified the interaction of genetic variation and dietary patterns on dyslipidemia in middle-aged Koreans.

In this study, we conducted to investigate the association between dietary pattern and GRS with the incidence of dyslipidemia in Korean adults using 14-years follow-up cohort data.

## 2. Materials and Methods

### 2.1. Subjects

The epidemiology data of the Ansung-Ansan cohort were obtained from the Korean Genome and Epidemiology Study (KoGES) conducted by the Korea Disease Control and Prevention Agency. The Ansung-Ansan cohort is an ongoing, prospective cohort study on Korean adults (40–69 years at baseline, *n* = 10,030) that began in 2001–2002. The follow-up examinations are conducted biennially. The cohort study design has been described in detail previously [19]. Informed consent was obtained from all study participants. The study protocol was approved by the Gachon University Institutional Review Board (1044396-201903-HR-041-03).

### 2.2. Methods

#### 2.2.1. Nutritional Assessment and Dietary Pattern Analysis

Dietary intake was assessed using validated 103-food items, semi-quantitative food frequency questionnaire (SQFFQ) [20]. The frequency of servings was classified into nine categories (never or seldom, 1 time/month, 2–3 times/month, 1–2 times/week, 3–4 times/week, 5–6 times/week, 1 time/day, 2 times/day, and 3 times or more/day). The size of the food item was classified into small, medium, and large. Among 10,030 participants, 9704 subjects had completed SQFFQ (96.8%). The nutrients intake of 103 food items was determined by the weight derived from the consumption frequency and portion size of each food items. The average nutrients intake of participants was calculated as the sum of the nutrient intake from each food item. The nutrient compositions used data from the seventh edition food composition tables of the Korean Nutrition Society [21].

To decrease the complexity, 103 food items were categorized into 36 groups, based on the food and nutrient composition similarity. Dietary patterns were derived using factor analysis based on the frequency of 36 food groups. The factors were rotated by an orthogonal transformation using the varimax rotation function to obtain a simpler structure and greater interpretability. To determine the number of factors, we considered eigenvalues >2 and inspection of scree plots. We decided to retain three major dietary factors.

All subjects had a dietary pattern score of three dietary patterns by weighing their intake of each food contributing to that pattern. Participants were divided into quartiles according to dietary pattern score of three dietary patterns.

#### 2.2.2. Definition of Dyslipidemia

The criteria for dyslipidemia followed the Clinical Practice Guideline of Korean Society of Lipid and Atherosclerosis [22]. Hypertriglyceridemia defined as elevated triglyceride (TG) level greater than 200 mg/dL and hypercholesterolemia defined as total cholesterol greater than 240 mg/dL or currently use any anti-dyslipidemic drug for controlling blood lipid level. Among participants who were not diagnosed or recognized with hypertriglyceridemia, participants with a fasting TG level >200 mg/dL in the health examination were considered to have hypertriglyceridemia [23]. Among 10,030 participants, we excluded subjects as follows: subjects without dietary data (*n* = 326), subjects without blood TG or total cholesterol information (*n* = 3), subject taking hypolipidemic medication (*n* = 57), subjects diagnosed dyslipidemia at baseline examination (*n* = 2214 for hypertriglyceridemia, *n* = 904 for hypercholesterolemia), and subjects didn’t attend the follow-up examination (*n* = 694 for hypertriglyceridemia, *n* = 840 for hypercholesterolemia). For the risk analysis of hypertriglyceridemia and hypercholesterolemia, a total of 6736 subjects and 7900 subjects were included, respectively.

#### 2.2.3. Measurement of Covariates

Height and weight were measured to the nearest 0.1 cm and 0.1 kg, respectively, by trained staff using a scale and a wall-mounted extensometer. Body mass index (BMI) was calculated as the weight in kilograms (kg)/height in meters (m) squared.

The general characteristics and lifestyle data were collected by an interviewer-administered questionnaire. We considered age, residual area (Ansung (rural) or Ansan (urban)), income (monthly household income; <1,500,000 won, and ≥15,000,000 won), education level (elementary school graduation or less, middle school graduation, high school graduation, and college graduation or higher), smoking status (current smoker or non-smoker), alcohol drinking status (current drinker or non/ex-drinker), regular exercise (yes or no), BMI, and total energy intake as potential confounding factors.

#### 2.2.4. Genotyping and GRS

The genotyping of KoGES participants has been described in detail previously [19,24]. In brief, genotyping of SNPs was conducted using the Affymatrix Genome-wide Human SNP array 5.0, and to improve the coverage, SNP imputation was performed using IMPUTE (v246) containing the 1000 Genomes Phase Ⅰ as a reference panel. Finally, 5,908,513 SNPs of 8840 participants were used in this analysis. Among them, 7 independent SNPs reported in a recent genome-wide association study (GWAS) of dyslipidemia [25,26,27] were selected for GRS. These SNPs that had a minor allele frequency in Asian >0.15 and have been robustly associated with dyslipidemia (*p* < 5 × 10^−7^). The selected seven SNPs were rs10889353 (*ANGPTL3*), rs7557067 (*APOB*), rs780092 (*GCKR*), rs1919127 (*C2orf16*), rs2954029 (*TRIB1*), rs174547 (*FADS1-FADS2-FADS3*), and rs2266788 (*APOA5*). We hypothesized an additive genetic model for each SNP applying a linear weight of 0, 1, or 2 to genotypes containing 0, 1, or 2 risk alleles, respectively, and the counted GRS of the seven selected SNPs was generated by summing the number of risk alleles for each SNP [28].

#### 2.2.5. Statistical Analysis

Descriptive statistics such as means values and standard deviation (SD) were used to summarize continuous variables and frequencies were expressed as percentages. The difference between groups was tested using the *t*-test for continuous variables and chi-square test for categorical variables. Multivariable-adjusted Cox proportional hazards regression model was conducted to compare the hazard ratios (HRs) and 95% confidence intervals (CIs) for dyslipidemia incidence risk according to the dietary pattern scores. Cox proportional hazards regression models were adjusted for age, sex, residual area, education level, household income, smoking status, alcohol drinking status, regular exercise, BMI, and total energy intake. To examine gene-diet interactions, we stratified subjects into tertile categories according to GRS and stratified participants into quartile categories according to “whole grain and soybean products” dietary pattern score. Then we performed stratified analyses to compare the relationship between dietary patterns and the HRs of dyslipidemia according to the GRS categories adjusted the same confounding factors as previous analysis. To test for trends, the factor scores entered into the model as continuous terms. A significant difference was defined as *p* < 0.05. All statistical analyses were performed using SAS 9.4 (SAS Institute Inc., Cary, NC, USA).

## 3. Results

### 3.1. Dietary Pattern Analysis

Three dietary patterns were identified from the factor analysis. Factor loadings and variances of each dietary pattern are shown in Table 1. Positive loading indicates that the dietary variable is positively associated with the factor and negative loading indicates an inverse association of the factor. The “meat, fish and vegetables” pattern featured a high consumption of green-yellow vegetables, root vegetables, meats, and fishes. The “bread and noodle” pattern had high positive factor loading for bread, noodles, and rice cake. The “whole grain and soybean products” pattern was characterized by high consumption of rice with grains (whole grain) and soybeans and soybean products whereas it had a negative loading for white rice and instant ramen.

### 3.2. Characteristics of Subjects by Each Dietary Pattern

Table 2 shows the general characteristics of participants by factor score quartiles for each dietary pattern. A highest factor score group for the “meat, fish and vegetables” pattern was younger, had higher BMI, higher energy intake, more male and more likely to live in an urban area, more educated, had higher income, and more regular exercise. Subjects with the higher score for the “Bread and noodle” pattern were younger, higher energy intake, higher total cholesterol and low TG. The highest quartile group of “whole grain and soybean products” pattern was older, had higher BMI and energy intake, higher total cholesterol and low TG, less educated, less likely to be a current smoker and drinker, and more likely to have regular exercise.

### 3.3. Analysis of Dyslipidemia Risk by Dietary Pattern

During the 14-year follow-up period, 2128 subjects (average follow up period, 8.50 years) and 2123 subjects (average follow up period, 8.34 years) were newly diagnosed with hypercholesterolemia and hypertriglyceridemia, respectively. Table 3 shows the analysis of dyslipidemia risk by dietary pattern score. After dividing the subjects into quartiles for each dietary pattern, the group with the highest factor score of “whole grain and soybean products” dietary pattern (rich in whole grain, soybeans, tofu, soybean paste, etc.) had a significantly lower risk of hypercholesterolemia (HR: 0.82, 95% CI: 0.72–0.93, *p* for trend = 0.0006) and hypertriglyceridemia (HR: 0.85, 95% CI: 0.75–0.97, *p* for trend = 0.0344) than the group with the lowest factor score after multi-adjusted confounding variables.

### 3.4. Interaction of Dietary Pattern with GRS on Dyslipidemia

Table 4 showed the characteristics of the seven selected SNPs. The minor allele frequency of the seven SNPs ranged from 0.17 to 0.47 and all SNPs were consistent with Hardy–Weinberg equilibrium.

The highest quartile of “whole grain and soybean products” dietary pattern group, compared with lowest dietary pattern group, had significantly lower risk of hypercholesterolemia (HR = 0.74, 95% CI: 0.59–0.93, *p* for trend = 0.0064) only among participants within the highest GRS tertile and GRS tertile 2 group also showed a tendency to reduce hypercholesterolemia risk (*p* for trend = 0.0433) (Table 5). But not among those with lower genetic risk. There was marginal interaction (*p*-interaction = 0.0815) between dietary patterns and GRS on the risk of hypercholesterolemia.

## 4. Discussion

In this study, three dietary patterns were identified using factor analysis, which are “whole grain and soybean products” pattern, “meat, fish and vegetables” pattern, and “bread and noodle” pattern. During 14-year follow-up period, the “whole grain and soybean products” pattern characterized high intakes of whole grain, soybeans, tofu, and soybean paste was related with a 15% and 18% lower risk of hypercholesterolemia and hypertriglyceridemia after adjustment for confounding factors, respectively. Other two dietary patterns were not related to dyslipidemia incidence. The “whole grain and soybean products” pattern is very similar to traditional Korean diet (K-diet) characterized with high consumption of vegetables, moderate to high consumption of legumes and fish, and seasoned with various *Jang* (fermented soy products), medicinal herbs, and sesame or perilla oil [29]. Dietary pattern analysis is widely used to examine the association between food intake and chronic diseases, because, they not only capture the complex of dietary intake but also explore the relationship with health outcomes [4]. In Western countries, the Mediterranean-style diet or DASH (dietary approaches to stop hypertension) are known to associate with dyslipidemia [30,31]. The vegetarian dietary patterns reduce the risk of CHD by about 40% in a meta-analysis of eight prospective cohort studies [32]. In a Framingham offspring cohort, subjects with a higher Mediterranean-style dietary pattern score (MSDPS) had significantly lower TG (*p* < 0.001) and higher high density lipoprotein (HDL) cholesterol (*p* = 0.02) after adjustment of confounding variables [33]. In the ATTICA study, a dietary pattern involving cereals, fish, legumes, vegetables, and fruits was inversely associated with TGs levels in Greece population [34].

In Korea, several previous studies conducted to examine the relationship between dietary pattern and dyslipidemia. In a cohort analysis, the prudent dietary pattern marked by high intakes of potatoes, legumes, vegetables, mushrooms, fish/shellfish and seaweed, reduced the low incidence of HDL-cholesterolemia to 0.76 in men and 0.78 in women, respectively [18]. In a cross-sectional study, the rice-oriented pattern was significantly increased prevalence of dyslipidemia in Korean adults men (OR = 1.58, *p* for trend = 0.0042, for hypertriglyceridemia, OR = 1.43, *p* for trend = 0.0015, for low HDL-cholesterol) and women (OR = 1.29, *p* for trend = 0.0020, for low HDL-cholesterol) using Korea National Health and Nutrition Examination Survey [35]. Another cross-sectional study showed that “oil, sweet, fish and other vegetables” dietary pattern and “grain, bean, nuts, vegetables and fruits” dietary pattern were lower risk of hypertriglyceridemia prevalence (OR = 0.73, *p* < 0.001 and OR = 0.88, *p* = 0.009, respectively) [7]. The Taiwan cross-sectional study showed similar results to our study, the vegetable-fruits-seafood dietary pattern (high intake of vegetables, vegetables with oil or dressing, fruits, seafood, legumes, soy products, and rice or flour products) decreased hypercholesterolemia risk (OR = 0.89) and dairy-complex carbohydrate dietary pattern (high intake of dairy products, milk, root crops, jam or honey, and whole-grain) decreased hypertriglyceridemia (OR = 0.82) in young and middle-aged Taiwanese [8].

A common feature of derived dietary patterns that lowers the risk of dyslipidemia is the high consumption of fiber-rich foods such as fruits, vegetables, and legumes. The whole grain and soybeans contained various nutrients such as dietary fiber, isoflavone, which help lower LDL cholesterol [36]. The soy protein has less hypercholesterolemia effect than casein, replacing dietary cholesterol with soy protein decreases the level of serum cholesterol in humans [37]. The insoluble fibers such as lignin, cellulose, and hemicellulose are rich in whole-grain foods, bran, nuts, and seeds. These fibers can be fermented and used by bacteria as a source of short-chain fatty acids. Therefore, absorption of short-chain fatty acids decreases cholesterol synthesis in the liver and reduces cholesterol levels in the blood [38].

Dyslipidemia is the combination result of the environmental (diet) factors and genetic factors [10]. After the human genome project completed, genome wide association studies have revealed many loci that may influence the lipid metabolism. However, the effect of individual SNPs on complex common disease is relatively weak [39]. Therefore, we examined the interaction between dietary patterns and genetic factors using GRS. The GRS has been used for combine the individual SNPs effects to reveal the complex relationship between CVD and genetic effects [12]. In the Framingham heart study, the 13 SNP GRSs were significantly associated with the incidence of hard CHD (HR = 1.07, *p* = 0.04) and CVD (HR for the allele, 1.05, *p* = 0.03) [14]. Another study used 4 SNP and subjects categorized into 3 GRS group (low 0–1, medium 2–3, and high 4–7), In high GRS group, high daily physical activity reduced the risk of MetS and low carbohydrate diet increased the risk of MetS [40].

In this study, we constructed GRSs composed 7 genetic variants (*ANGPTL3* rs10889353, *APOB* rs7557067, *GCKR* rs780092, *C2orf16* rs1919127, *TRIB1* rs2954029, *FADS1-FADS2-FADS3* rs174547, and *APOA5* rs2266788) associated with dyslipidemia using GWAS studies [25,26,27]. To summarize the characteristics of genes [41], *ANGPTL3* (Angiopoietin Like 3) is a protein coding gene and associated diseases were familial hypobetalipoproteinemia 1 and 2 which related to the lipoprotein metabolism pathway. *APOB* (Apolipoprotein B) is a protein coding gene and associated disorder were familial hypobetalipoproteinemia 1 and 2 associated with activated Toll-like receptor (TLR) 4 signaling and lipoprotein metabolism pathway. *TRIB1* (Tribbles Pseudokinase 1) is a protein coding gene and related ailments were megakaryocytic leukemia and familial hypercholesterolemia. *APOA5* (Apolipoprotein A5) is a protein-coding gene and linked disorders were hyperlipoproteinemia Type V and familial hypertriglyceridemia, lipoprotein metabolism, and lipid signaling pathway. *FADS1* (Fatty Acid Desaturase 1) is a protein-coding gene and associated with lipid metabolism disorder and mainly expressed in the liver, and catalyze the desaturation steps in the synthesis of n-3 and n-6 polyunsaturated fatty acids. The *ANGPTL3* rs10889353, *APOB* rs7557067, *TRIB1* rs2954029, and *FADS1-FADS2-FADS3* rs174547 were significantly associated with TG level in meta-analysis of seven GWASs [25] and *FADS1-FADS2-FADS3* rs174547 C allele was significantly increased TG level in the Japanese population [42]. Recently, genes associated with high TG level in Koreans were identified as the *GCKR* rs780092, *C2orf16* rs1919127 and *APOA5* rs2266788 [27] and the association between rs2266788 in the *APOA5* gene and high TG level also reported in Chinese population [43].

We found that the “whole grain and soybean products” dietary pattern related with counted GRS to modulate the risk of the hypercholesterolemia risk in Korean adults. In the high GRS group, the highest quartile of “whole grain and soybean products” dietary pattern score decreased 26% hypercholesterolemia risk (*p* for trend = 0.0064) compared with the lowest quartile. These results suggest that the effect of dietary patterns in reducing the risk of hypercholesterolemia is more effective in the group with higher GRS level. In Framingham Heart Study, high GRS level were positively associated with liver fat accumulation, than those who had low Mediterranean diet score and Alternative Health Eating Index (AHEI) [44]. Furthermore, in a Sweden prospective cohort study, diet quality (based on intakes of saturated fat, polyunsaturated fat, sucrose, fiber, fruit and vegetables, and fish) and HDL-cholesterol change were associated with lower GRS group only [16]. In the PREDIMED-NAVARRA Randomized Trial, Pro/Ala (rs1801282) in the peroxisome proliferator-activated receptor gamma isoform 2 (PPARγ2) was associated with telomere length, especially in the group with high adherence to the Mediterranean diet pattern [45]. These results indicate that the effects of dietary factors on disease vary with individual genotypes. Therefore, personalized nutrition therapy is needed considering personal genotype.

This study has several limitations. First, participants of the study lived in a small residual area and included only middle-aged and older adults. Second, the GRS relied upon only seven SNPs discovered from the findings of GWAS analysis which could describe the small portions of dyslipidemia. However, we considered theses minor allele frequency of these SNPs and significances of the analyses and selected the most significant SNP in the same linkage disequilibrium (LD) block.

Also, this study had several strengths. First, we examine the causal relationship between dietary patterns, GRS and dyslipidemia incidence risk using 14-years follow-up prospective cohort study. Second, the three dietary patterns were estimated using a validated SQFFQ collected dietary consumption data for more than 1 year, therefore, this FFQ reflecting long-term dietary intake of participants more than the 24-hr recall.

## 5. Conclusions

In conclusion, for people with genotypes that can cause hypercholesterolemia, eating whole grains and soybean products may have a meaningful response. The results of this study are expected to be utilized for genome-based precision nutrition therapy to treat and prevent dyslipidemia.

## Figures and Tables

**Table 1:** Factor loading * matrix for 3 dietary patterns from the food frequency questionnaire.

Food Groups	Meat, Fish and Vegetables	Bread and Noodle	Whole Grain and Soybean Products
White rice			−0.83
Rice with grains			0.85
Ramen		0.40	−0.21
Noodles		0.39	
Other noodles		0.61	
Dumplings		0.61	
Rice cake		0.57	
Bread		0.66	
Pizza/Hamburger		0.37	
Flake		0.32	
Cakes		0.36	
Snacks/Sweets		0.24	
Butter/Margarine		0.31	
Potatoes	0.31	0.45	
Soybeans and beans products	0.24		0.36
Nuts	0.15	0.24	
Kimchi	0.25		
Green-yellow vegetables	0.61		
Root vegetables	0.62		
Pickle/Salt-fermented fish	0.37		
Mushrooms	0.52		
Fruits	0.48		
Meats	0.64		
Eggs	0.37		
White fish	0.62		
external blue colored fish	0.59		
Anchovy	0.39		0.24
Cuttlefish/Octopus	0.52		
Fish cake	0.39		
Shellfish	0.55		
Seaweeds	0.44		0.21
Milk			0.23
Dairy products	0.21		0.24
Carbonated drinks			
Coffee			
Green tea and other drinks	0.23		
Variance explained	4.21	2.87	2.03

* factor loading coefficient greater than 0.2 are shown for simplicity.

**Table 2:** Baseline characteristics of the study participants across quartile (Q) of dietary pattern scores.

	Meat, Fish and Vegetables		Bread and Noodle		Whole Grain and Soybean Products	
	Q1 (*n* = 2426)	Q4 (*n* = 2426)	Q1 (*n* = 2426)	Q4 (*n* = 2426)	Q1 (*n* = 2426)	Q4 (*n* = 2426)
Mean ± SD	Q1	Q4	Q1	Q4	Q1	Q4
Age	54.9 ± 0.2	50.7 ± 0.2 ***	55.2 ± 0.2	49.7 ± 0.2 ***	51.3 ± 0.2	53.7 ± 0.2 ***
BMI	24.4 ± 0.1	24.8 ± 0.1 ***	24.6 ± 0.1	24.6 ± 0.1	24.4 ± 0.1	24.7 ± 0.1 ***
Energy	1585.4 ± 11.5	2472.9 ± 17.4 ***	1720.8 ± 13.4	2412.4 ± 16.6 ***	1918.4 ± 15.0	2050.3 ± 13.6 ***
Total cholesterol	190.7 ± 0.7	192.0 ± 0.7	190.0 ± 0.7	192.7 ± 0.7 *	191.1 ± 0.7	193.5 ± 0.8 **
HDL cholesterol	44.6 ± 0.2	44.7 ± 0.2	44.2 ± 0.2	45.1 ± 0.2 **	44.6 ± 0.2	45.1 ± 0.2
Triglyceride	162.5 ± 2.1	162.7 ± 2.2	165.1 ± 2.0	163.4 ± 2.3	167.2 ± 2.2	158.1 ± 2.2 *
Male (%)	43.2	49.5 ***	40.5	51.4 ***	62.9	34.0 ***
Area (%)						
Rural	64.5	45.3 ***	62.2	39.9 ***	58.8	40.0 ***
Urban	35.5	54.7	37.8	60.1	41.2	60.0
Education (%)						
Elementary school	48.7	25.3 ***	49.3	21.3 ***	32.9	35.2 ***
Middle school	21.3	23.2	22.9	20.8	24.0	21.6
High school	22.2	34.7	20.9	37.7	30.5	29.2
College or higher degree	7.8	16.8	6.9	20.3	12.6	14.0
Income (%)						
<1,500,000 won	66.5	41.7 ***	64.7	40.1***	54.0	49.4 ***
≥1,500,000 won	33.5	58.3	35.3	59.9	46.0	50.6
Current drinking (%)						
Yes	40.9	51.0 ***	41.9	51.5 ***	56.1	38.5 ***
Current smoking (%)						
Yes	17.2	18.8	14.8	20.2 ***	21.1	16.1 ***
Regular exercise (%)						
Yes	21.0	33.9 ***	22.3	33.2 ***	21.2	35.2 ***

SD: standard deviation, HDL: high-density lipoprotein; BMI: body mass index. * *p* < 0.05, ** at *p* < 0.01 and *** at *p* < 0.001.

**Table 3:** Hazard ratios (HR) and 95% confidence interval (CI) for the risk of incident dyslipidemia according to dietary pattern score.

		No of Total	No of Cases	Model 1		Model 2	
				HR 95% CI		HR 95% CI	
**Hypercholesterolemia**							
Meat, fish and vegetables	Q1	1967	530	1.00		1.00	
	Q2	1973	533	1.02	0.90–1.15	0.92	0.81–1.04
	Q3	1977	548	1.06	0.94–1.19	0.95	0.83–1.08
	Q4	1983	517	1.02	0.90–1.15	0.91	0.79–1.05
*p* for trend				0.7574		0.3079	
Bread and noodle	Q1	1992	520	1.00		1.00	
	Q2	1986	538	1.05	0.93–1.18	1.03	0.91–1.17
	Q3	1968	525	1.03	0.91–1.17	0.98	0.86–1.11
	Q4	1954	545	1.11	0.98–1.26	1.05	0.92–1.20
*p* for trend				0.1071		0.556	
Whole grain and soybean products	Q1	1985	506	1.00		1.00	
	Q2	1986	532	0.98	0.87–1.11	0.93	0.82–1.06
	Q3	2000	545	0.94	0.83–1.06	0.83	0.73–0.94
	Q4	1929	545	0.94	0.83–1.06	0.82	0.72–0.93
*p* for trend				0.2246		0.0006	
**Hypertriglyceridemia**							
Meat, fish and vegetables	Q1	1691	532	1.00		1.00	
	Q2	1672	521	1.00	0.89–1.13	0.99	0.87–1.12
	Q3	1695	556	1.05	0.93–1.18	1.03	0.90–1.16
	Q4	1678	514	1.01	0.89–1.14	1.00	0.87–1.16
*p* for trend				0.806		0.8508	
Bread and noodle	Q1	1661	543	1.00		1.00	
	Q2	1704	564	0.97	0.86–1.09	0.96	0.86–1.09
	Q3	1724	522	0.87	0.77–0.99	0.87	0.77–0.99
	Q4	1647	494	0.86	0.76–0.98	0.87	0.76–1.00
*p* for trend				0.0126		0.0363	
Whole grain and soybean products	Q1	1653	558	1.00		1.00	
	Q2	1670	503	0.87	0.77–0.98	0.87	0.77–0.98
	Q3	1681	524	0.88	0.78–0.99	0.85	0.75–0.97
	Q4	1732	538	0.87	0.77–0.98	0.85	0.75–0.97
*p* for trend				0.0613		0.0344	

Model 1 adjusted for age and sex, Model 2 adjusted for age, sex, residual area, education level, household income, current drinking, current smoking, physical activity, total energy intake and BMI, Q: quartile.

**Table 4:** Characteristics of the seven selected single-nucleotide polymorphisms (SNPs).

SNP Name	Chromosome	Position	Gene	P-HWE	MAF(ASN)	Ref Allele	Alt Allele	Risk Allele	*p*-Value	Source
rs10889353	1	63118196	*ANGPTL3*	0.2673	0.173	A	C	C	3.00 × 10^−7^	PMID: 19060906
rs7557067	2	21208211	*APOB*	0.4536	0.272	A	G	G	9.00 × 10^−12^	PMID: 19060906
rs780092	2	27743154	*GCKR*	0.5279	0.326	A	G	A	2.00 × 10^−9^	PMID: 23105936
rs1919127	2	27801493	*C2orf16*	0.4481	0.474	T	C	T	3.00 × 10^−11^	PMID: 31910446
rs2954029	8	126490972	*TRIB1*	0.0774	0.442	A	T	A	3.00 × 10^−19^	PMID: 19060906
rs174547	11	61570783	*FADS1-FADS2-FADS3*	0.0739	0.322	T	C	C	2.00 × 10^−14^	PMID: 19060906
rs2266788	11	116660686	*APOA5*	0.5322	0.218	G	A	A	2.00 × 10^−16^	PMID: 31910446

HWE: Hardy–Weinberg equilibrium, MAF: minor allele frequency, ASN: Asian, Ref: reference, Alt: alternate.

**Table 5:** Hazard ratios (HR) and 95% confidence intervals (CI) of dyslipidemia risk according to the joint classifications of the whole grain and soybeans products dietary pattern score and genetic risk scores (GRS) categories.

GRS Categories	Whole Grain and Soybean Products Dietary Pattern Score	Model 1			Model 2		
		HR	95% CI	*p*	HR	95% CI	*p*
**Hypercholesterolemia**							
GRS Tertile 1	Q1	1.00			1.00		
	Q2	1.20	0.92–1.56	0.1857	1.12	0.86–1.48	0.3999
	Q3	1.22	0.94–1.58	0.1365	1.08	0.83–1.42	0.5537
	Q4	1.11	0.85–1.44	0.4559	1.00	0.76–1.32	0.9824
	*p* for trend	0.583			0.8119		
GRS Tertile 2	Q1	1.00			1.00		
	Q2	1.13	0.92–1.40	0.2527	1.05	0.85–1.30	0.6688
	Q3	1.07	0.86–1.32	0.5425	0.88	0.71–1.09	0.2407
	Q4	1.07	0.86–1.33	0.558	0.85	0.68–1.07	0.1722
	*p* for trend	0.8734			0.0433		
GRS Tertile 3	Q1	1.00			1.00		
	Q2	0.84	0.68–1.04	0.0995	0.81	0.65–1.01	0.0577
	Q3	0.74	0.59–0.92	0.0066	0.68	0.55–0.86	0.0009
	Q4	0.82	0.66–1.02	0.0707	0.74	0.59–0.93	0.011
	*p* for trend	0.053			0.0064		
*p*_interaction_ = 0.0815							
**Hypertriglyceridemia**							
GRS Tertile 1	Q1	1.00			1.00		
	Q2	0.99	0.77–1.28	0.95	1.04	0.80–1.34	1.232
	Q3	1.06	0.83–1.35	0.66	1.04	0.81–1.33	1.175
	Q4	0.92	0.71–1.17	0.482	0.92	0.71–1.20	1.251
	*p* for trend	0.6411			0.5676		
GRS Tertile 2	Q1	1.00			1.00		
	Q2	0.90	0.72–1.11	0.3236	0.88	0.71–1.10	0.2547
	Q3	0.92	0.74–1.13	0.415	0.87	0.70–1.08	0.1976
	Q4	1.01	0.82–1.24	0.9506	0.97	0.77–1.21	0.7566
	*p* for trend	0.788			0.8376		
GRS Tertile 3	Q1	1.00			1.00		
	Q2	0.84	0.68–1.05	0.1217	0.87	0.70–1.08	0.2104
	Q3	0.80	0.64–1.00	0.0535	0.81	0.64–1.02	0.0725
	Q4	0.83	0.67–1.04	0.0992	0.87	0.69–1.10	0.2556
	*p* for trend	0.1299			0.2514		
*p*_interaction_ = 0.5023							

Model 1 adjusted for age and sex, Model 2 adjusted for age, sex, residual area, education level, household income, current drinking, current smoking, physical activity, total energy intake and BMI, Q: quartile.
